# Practical Considerations for Use of Direct Oral Anticoagulants in Children

**DOI:** 10.3389/fped.2022.860369

**Published:** 2022-04-01

**Authors:** Hilary Whitworth, Leslie Raffini

**Affiliations:** Children's Hospital of Philadelphia, Philadelphia, PA, United States

**Keywords:** pediatric, thrombosis, VTE, anticoagulation, direct oral anticoagulant

## Abstract

Direct oral anticoagulants (DOACs) provide an attractive alternative for the management and prevention of thrombosis in pediatric patients. With multiple ongoing and published pediatric trials and recent regulatory approval of dabigatran and rivaroxaban, the landscape of pediatric anticoagulation is rapidly changing. However, as pediatricians gain experience with these drugs, it is important to be mindful of pediatric-specific considerations that may limit the use of DOACs in certain children and adolescents. While there is increasing adult data and experience, there is a paucity of real-world evidence to guide the use of these drugs in children who would not have met clinical trial inclusion criteria. In this mini review, we summarize pediatric specific data, areas for future research, and practical considerations for the use of DOACs in children and adolescents.

## Introduction

The rate of venous thromboembolism (VTE) in children continues to increase, mostly in hospitalized children with complex medical problems (1). Until recently, anticoagulation has been limited to unfractionated heparin (UFH), low molecular weight heparin (LMWH), and vitamin K antagonists (VKA), which all have drawbacks—including the need for intravenous access, subcutaneous injections, and/or frequent monitoring (2). Direct oral anticoagulants (DOACs) which inhibit factor Xa or thrombin do not require monitoring and have become preferred over VKA for treatment of VTE in adults (2). The use of DOACs in children is increasing as pediatric clinical trial data emerge and regulatory approvals are submitted or completed (3–11). In addition to treatment of pediatric VTE, DOACs are also being investigated for prevention of thrombosis in children with congenital heart disease and cancer (6–9).

As of January 2022, the DOACs with the most published pediatric data are rivaroxaban and dabigatran, and both have recent approval for treatment of pediatric VTE by the European Medicines Agency (EMA) and US Food and Drug Administration (FDA). The EINSTEIN-Junior trial compared the direct Xa inhibitor rivaroxaban to standard of care (SOC) after 5–9 days of a parenteral anticoagulant in 500 patients (birth to 18 years) with VTE. Rates of recurrent VTE and bleeding in children were similarly low in both groups (5). The DIVERSITY trial compared the direct thrombin inhibitor dabigatran to SOC after 5–21 days of a parenteral anticoagulant in 328 patients (birth to 18 years) with VTE (4). This study showed dabigatran to be non-inferior to SOC for the primary composite endpoint of thrombus resolution, freedom from recurrent VTE, and freedom from VTE-related death without increase in bleeding events (4). There are ongoing pediatric clinical trials to evaluate apixaban and edoxaban, additional direct Xa inhibitors that are FDA approved in adults (10, 11).

While available trial data are encouraging and offer new options for many children with VTE, there remain many unanswered questions (Table 1). In this mini review, we discuss how recent data support the use of DOACs in specific pediatric populations (Table 2), and highlight situations where DOACs may not be the best option and/or more data is necessary. When relevant, we include informative adult studies. This is a rapidly evolving field with several ongoing clinical trials, and as new data emerge, these recommendations will need to be updated.

**Table 1:** Priorities for additional research regarding use of DOACs in children.

• Treatment of VTE: additional prospective data (safety and efficacy) on infants and younger children
• Pharmacokinetic and pharmacodynamics of DOAC reversal agents in children
• Role of DOAC monitoring in specific populations for adherence, safety, or reversal
• Use of DOACs for children with arterial thrombosis (catheter related and stroke)
• Menorrhagia in adolescents with DOACs
• Periprocedural protocols
• Adherence to DOACs vs SOC

**Table 2:** Considerations for initiating DOACs for treatment of pediatric VTE.

• Stable patient (unlikely to need an urgent procedure)
• Tolerating good oral intake or on full nasogastric feeds
• Adequate renal and hepatic function
• Unlikely to have antiphospholipid antibody syndrome
• ≥5 days of parenteral anticoagulation
• No drug interactions (consult pharmacy)
• Gestational age > 37 weeks and weight > 2.6 kg
• Availability of appropriate drug formulation

## Treatment of VTE

### Acute VTE

Most children who develop VTE have complex chronic conditions, multiple risk factors, and are hospitalized. Advantages of DOACs, including no monitoring and oral administration, are potential disadvantages in hospitalized children whose clinical status may change quickly, necessitating unplanned procedures or discontinuation of oral intake. The DOAC trials required that children with acute VTE receive a parenteral anticoagulant (UFH or LMWH) for at least 5 days prior to initiating the DOAC (4, 5). This is different from adult regimens, where DOACs (apixaban and rivaroxaban) can be used as initial treatment for acute VTE at a higher dose intensity for 1–3 weeks (12–14). Initial DOAC therapy for pediatric VTE may be desired in some situations, such as clinically stable, older adolescents with low likelihood of antiphospholipid antibody syndrome (APS), and can be considered (off label) in patients > 50 kg, based on adult regimens. Whether initial DOAC therapy is safe and effective for acute VTE in clinically stable younger children, and if higher intensity is needed, remains unknown.

### Age

The bimodal age peaks of VTE in children < 1 year of age and adolescents have been long recognized (1, 15, 16). Children < 1 year are underrepresented in the DOAC pediatric investigational programs due to inclusion/exclusion criteria and the challenges of enrolling young children on interventional trials. Trials restricted enrollment to neonates with gestational age > 37 weeks and/or body weight of at least 2.6 kg (4, 5). Oral formulations appropriate for infants are not available and limit their current use outside of clinical trials. Additional data on infants from prospective observational studies will be important to confirm the results observed in the small cohorts included in the clinical trials.

### Central Venous Catheter Related Thrombosis (CVC-VTE)

While the majority (80–90%) of pediatric VTE is related to a central venous catheter (CVC), patients with CVC-VTE represent a minority (15–27%) in pediatric DOAC trials (4, 5, 17). This may be because children who develop CVC-VTE are often acutely ill and may not be good candidates for a DOAC. However, sub analysis of 126 children with CVC-VTE in the EINSTEIN-Jr trial (90 rivaroxaban, 36 SOC) identified no recurrent VTE or major bleeding in either study arm, suggesting that DOACs are safe and effective for patients who met inclusion criteria (18).

### Cerebral Venous Thrombosis (CVT)

CVT is often associated with risk factors such as cancer, head and neck infections, thrombophilia, and hormonal contraception (19, 20). Although use of anticoagulants in CVT must be balanced with the risk of intracranial hemorrhage, therapeutic anticoagulation is generally recommended in patients without hemorrhage (2). In a sub analysis of 114 children with CVT in the EINSTEIN-Jr trial (73 rivaroxaban, 41 SOC), symptomatic recurrent VTE at 3 months was rare (none in the rivaroxaban group, 1 in SOC group) (21). There were no major bleeding events in either cohort; clinically relevant non-major bleeding (CRNM) was observed in 5/73 (6.8%) of the rivaroxaban recipients compared to 1/41 in the SOC group (21). All patients were treated with LMWH or UFH for 5–9 days prior to randomization (21).

### Malignancy

Children with cancer have multiple risk factors for VTE, including the cancer itself, CVCs, and prothrombotic medications including asparaginase and steroids (22). Anticoagulation can be challenging in oncology patients as they are frequently thrombocytopenic, require repeated procedures, have variable oral intake, and require medications that cause drug–drug interactions. The published pediatric DOAC trials did include children with cancer although excluded those with thrombocytopenia (platelet count <50–80 × 10^9^/L) or considered to be at a high risk of bleeding (4, 5). EINSTEIN-Jr included 40 patients with active cancer in the rivaroxaban arm and 16 patients in the SOC arm (5). DIVERSITY included 1 patient with active or previous cancer in the SOC arm and 18 in the dabigatran arm (4). These patients were not analyzed separately, yet overall rates of recurrent VTE and bleeding were low in both trials.

Meta-analysis of several large clinical trials comparing DOACs to LMWH in adults with cancer has concluded that DOACs are an effective treatment option for adults with cancer but should be used with caution in patients at high risk of bleeding (23). 2021 ASH guidelines suggest DOACs over LMWH for adults with cancer (24). Use of DOACs in children with cancer and VTE is reasonable, although there is limited data on management around procedures and thrombocytopenia, and drug interactions may be more frequent in this population.

### Cardiac Disease

Children with congenital and acquired heart disease often have unique risk factors for thrombosis including abnormal vascular flow, surgery, venous and arterial catheters, coagulation factor imbalances, and intracardiac devices. EINSTEIN-Jr included 35 children with cardiac disease in the rivaroxaban arm and 14 patients in the SOC arm (5). In the DIVERSITY study, 21 patients with congenital heart disease and VTE were included in the dabigatran arm and 27 patients in the SOC arm (4).

### Obesity

Current International Society of Thrombosis and Hemostasis guidelines for adults with VTE who are >120 kg or BMI > 40 kg/m^2^ suggest that standard doses of rivaroxaban or apixaban are appropriate options regardless of high BMI and weight but suggest the other DOACs continue to be avoided in this population due to the lack of data (25). Although patients > 120 kg were not included in the pediatric clinical trials, it is reasonable to extrapolate adult data to patients < 18 years of age who are >120 kg and are otherwise acceptable candidates for DOACs.

### Renal Impairment

Each of the DOACs requires some degree of renal excretion which is highly variable across drugs. Dabigatran is the most reliant on renal excretion and apixaban is the least (renal excretion by drug: apixaban 27%, rivaroxaban 36%, dabigatran 80%, and edoxaban 50%) (26). Patients with renal dysfunction may have decreased clearance and increased risk of bleeding complications. The DIVERSITY and EINSTEIN-Jr studies excluded children with severe renal dysfunction. Patients with estimated glomerular filtration rate (eGFR) < 50 mL/min/1.73 m^2^ (Schwartz formula) or on dialysis were excluded from DIVERSITY while children with eGFR <30 mL/min/1.73 m^2^ and children <1 year old with serum creatinine > 97.5th percentile were excluded from EINSTEIN-Jr (4, 5).

Use of DOACs in adults with various degrees of renal insufficiency and renal failure continues to evolve as more data are available (27). Dose reductions and recommendations based on renal clearance for adults are provided in the package insert for each drug.

### Liver Disease

Children with abnormal liver function studies were also excluded from the DOAC trials. In EINSTEIN-Jr, exclusion criteria included ALT > 5× upper limit of normal (ULN) or bilirubin > 2× ULN; in DIVERSITY, those with ALT, AST or alkaline phosphatase >3× ULN were excluded (4, 5). In general, DOACs are not recommended for adults with moderate or severe hepatic impairment due to associated coagulation abnormalities and should also be avoided in children who would have been excluded based on above criteria until further data is available.

### Antiphospholipid Antibody Syndrome

Adult data show inferiority of factor Xa inhibitors compared to VKA in patients with APS, particularly those with triple positive antiphospholipid antibodies (APA) (28–30). The ASH 2020 guidelines for management of VTE in adults exclude APS in their suggestion for DOACs over VKAs (31). Currently, the FDA labels for all DOACs exclude patients with triple positive APA. Most of the data leading to these recommendations is from studies evaluating rivaroxaban compared to VKAs, although one meta-analysis did include 144 patients treated with dabigatran (30). The DIVERSITY trial included 11 children with APA, 7 in the SOC arm and 4 in the dabigatran arm (4). Detailed information about these patients or their APA titers was not provided. Given the current literature, we would not recommend using DOACs in children with APS. Whether or not to test children with VTE for APA prior to use of a DOAC depends on the clinical scenario, recognizing that DOACs often cause a false positive dilute Russel Viper Venom Time (32).

### Abnormal Uterine Bleeding (AUB)

Emerging data evaluating menstrual bleeding in adults indicates increased rates of AUB in women on rivaroxaban compared to VKAs and apixaban (33, 34). EINSTEIN-Jr reported menorrhagia in 7% of the rivaroxaban arm and 3% of SOC arm, none of which met criteria for major or CRNM bleeding (5). AUB was not separately described in the DIVERSITY study (4). These data are limited by variable definitions of AUB and denominators that may include all participants instead of only menstruating participants. This area deserves further investigation, particularly in female adolescents with VTE.

## Prevention of Thrombosis

Evidenced based guidelines for prevention of thrombosis in children are lacking. Nonetheless, underlying conditions including cancer and congenital heart disease are associated with a substantial risk of thrombosis. There are several DOAC trials investigating thromboprophylaxis in these populations, but the trials are underpowered to demonstrate efficacy.

### Cardiac Disease

The UNIVERSE study was a randomized trial comparing rivaroxaban to aspirin for thromboprophylaxis in 112 children aged 2–8 years, within 4 months after a Fontan procedure (6). Rivaroxaban dosing in this study was targeted to achieve equivalent to 10 mg daily dosing in adults (6). The rate of thrombotic events was 3% (2/76) of the rivaroxaban group and 9% (3/34) in the aspirin group, with low rates of bleeding in both groups (6). Based on these data, rivaroxaban received FDA approval for thromboprophylaxis in children >2 years old with Fontan physiology.

ENNOBLE-ATE is a randomized trial to evaluate the safety of edoxaban compared to LMWH or VKA for thromboprophylaxis in children with cardiac diseases at risk for thromboembolism including patients with Fontan physiology, Kawasaki disease, heart failure, or other cardiac indication for thromboprophylaxis (7). The SAXOPHONE trial is a randomized trial comparing apixaban to LMWH or VKA to prevent thrombosis in children and infants with congenital and acquired heart disease (8). ENNOBLE-ATE and SAXOPHONE target therapeutic dose intensity DOACs which differs from UNIVERSE, which targeted prophylactic intensity rivaroxaban (6–8). These studies will inform thromboprophylaxis strategies in children with cardiac disease.

### Oncology

Current guidelines do not recommend routine thromboprophylaxis for children with cancer, although some hospitalized children and adolescents with multiple VTE risk factors may be started on prophylaxis. The PREVAPIX-ALL trial is a recently completed randomized trial designed to evaluate the efficacy and safety of apixaban for thromboprophylaxis in children with acute lymphoblastic leukemia or T or B cell lymphoblastic lymphoma and a CVC receiving asparaginase therapy (9). Apixaban, at equivalent dose intensity used for adult VTE prophylaxis (2.5 mg twice daily) was compared to no anticoagulation (9). Patients at high risk for bleeding were excluded, including those requiring multiple lumbar punctures (LPs), known bleeding disorder or coagulopathy, uncontrolled hypertension, hyperleukocytosis, or major surgery within 7 days of enrollment (9). Patients were required to have a platelet count ≥20,000/uL within 24 h of starting treatment and apixaban was held when the platelet count was <20,000/uL (9). The anticipated result of this study, which includes 512 children, will inform future thromboprophylaxis strategies for children with leukemia and a CVC.

### Acute Ischemic Stroke (AIS)

DOACs are routinely used and approved to prevent AIS in adults with atrial fibrillation. There have not been any pediatric studies investigating the role of DOACs for primary or secondary AIS prevention. The etiology of AIS in children is multifactorial, however up to 45% have intracranial arteriopathy which is associated with a high risk of recurrent stroke (35). While both antiplatelet therapy and anticoagulants are used to prevent recurrent stroke in children, it is not always clear which approach is best and therapy is individualized by pediatric stroke experts (35).

## Route of Administration and Formulation

New oral formulations (e.g., suspension, pellets, or sprinkles) of each DOAC had to be developed and investigated. Although anticipated soon, none of these formulations are currently available. While rivaroxaban and apixaban tablets can be crushed and mixed with applesauce immediately prior to use or with water and administered *via* nasogastric tube, dabigatran capsules cannot be crushed and must be swallowed whole (36).

## Medication Interactions

Medication interactions occur far less frequently with DOACs than VKA. The most common interactions are mediated by medications that induce or inhibit cytochrome P450 enzyme or permeability glycoprotein (37). CYP3A4 is important for the metabolism of apixaban and rivaroxaban but not the other DOACs (37). Drugs that may affect these pathways and are commonly used in children include rifampin, carbamazepine, phenytoin, phenobarbital, clarithromycin, amiodarone, fluconazole, and cyclosporine (37). It is good practice to consult with a pharmacist about potential drug interactions prior to prescribing a DOAC.

## Bleeding/Reversal/Procedures

Bleeding remains the most serious complication of anticoagulants. Rates of major bleeding in the DOAC clinical trials have been low: 2% in the dabigatran trial and none in the rivaroxaban trial (4, 5). While reassuring, it is important to have a plan for addressing major bleeding in children receiving DOACs. Targeted reversal agents for DOACs are available for use in adults, but have not been studied in children. In patients with non-life-threatening bleeding, supportive care (holding anticoagulant and transfusions) is often sufficient.

Dabigatran is 35% protein bound so can be partially cleared by dialysis or hemofiltration (38). Idarucizumab is a humanized monoclonal antibody fragment that binds to dabigatran and its metabolites with higher affinity than the binding affinity of dabigatran to thrombin, neutralizing the anticoagulant effect immediately after administration (39). Idarucizumab is FDA approved for reversal of dabigatran in adults with life threatening bleeding or for emergent procedures. Published pediatric data is limited to a case report of a 15-year-old, 67 kg female who received idarucizumab without adverse effects after a dabigatran overdose (40).

Factor Xa inhibitors are protein bound and cannot be cleared by dialysis (41). Andexanet alfa works as an inactive recombinant human factor Xa to compete with active factor Xa binding to apixaban and rivaroxaban and is approved for reversal in adults on rivaroxaban or apixaban with life threatening bleeding (42). Published pediatric data is limited to a case report of a 4-year-old, 20 kg female who received andexanet alfa due to an intracranial hemorrhage while taking rivaroxaban without adverse effects (43).

In addition to the lack of safety and efficacy data in pediatrics, the use of these reversal agents is limited by cost and availability, even in adult hospitals. Pediatric hospitals may not have access to these medications or may need to work with an adult hospital to send the medication, which is not ideal for life-threatening bleeding. Prothrombin complex concentrates have been used in the setting of hemorrhage with oral factor Xa inhibitors but are not approved for this indication in adults or pediatrics (44).

Management of DOACs around procedures has not been studied in children. In the Perioperative Anticoagulation Use for Surgery Evaluation (PAUSE) study, rates of major bleeding in adults with atrial fibrillation receiving apixaban, dabigatran, or rivaroxaban were low using a standardized protocol (45). In this protocol, DOACs were held for 1 day for “low- bleeding risk” procedures and 2 days for “high-bleeding risk” procedures (45). In the PREVAPIX-ALL study, apixaban was held for 24 h prior to LPs and at least 18–24 h after the procedure and pharmacokinetic and pharmacodynamic data were collected (9). While this may provide some evidence to guide periprocedural management, this study used prophylactic dose intensity, so these data are not generalizable to children receiving therapeutic dosing.

## Discussion

New evidence to support DOACs for treatment and prevention of thrombosis in children is changing the therapeutic landscape. DOACs offer great advantages over standard anticoagulants for many, but not all, children with thrombosis. Whether these advantages will translate to improved adherence and outcomes is unknown. While current phase III clinical trial data support safety and efficacy for treatment of acute pediatric VTE, there remain many unanswered questions around use of DOACs in lieu of standard anticoagulants for children who would not have met trial inclusion criteria. It is currently not possible to make evidence-based recommendations for use in children who would not have met inclusion criteria. There are several ongoing trials (mentioned above) that will provide additional data in the upcoming years, but additional prospective cohort studies will be necessary to fill in the many gaps (Table 1).

## Author Contributions

HW and LR contributed to the writing and review of the manuscript and approved the final submission. All authors contributed to the article and approved the submitted version.

## Funding

HW was supported by National Institutes of Health, National Heart, Lung, and Blood Institute grant T32 HL007971.

## Conflict of Interest

LR has received consulting fees from Boehringer Ingelheim. The remaining author declares that the research was conducted in the absence of any commercial or financial relationships that could be construed as a potential conflict of interest.

## Publisher's Note

All claims expressed in this article are solely those of the authors and do not necessarily represent those of their affiliated organizations, or those of the publisher, the editors and the reviewers. Any product that may be evaluated in this article, or claim that may be made by its manufacturer, is not guaranteed or endorsed by the publisher.
